# Establishing a Predictive Model for the Occurrence of CI-AKI After PCI in Patients With Coronary Heart Disease Based on Serum-Derived Biomarkers

**DOI:** 10.1155/crp/9997784

**Published:** 2025-07-27

**Authors:** Qin-yu Sun, Min-jia Tang, Lin Shi, Yi-fan Deng, Zhen Fang, Jun Ji, Sheng-hu He, Jing Zhang

**Affiliations:** ^1^Department of Cardiology, Northern Jiangsu People's Hospital Affiliated to Yangzhou University, Yangzhou 225001, Jiangsu, China; ^2^Department of Medicine, Yangzhou University, Yangzhou 225001, Jiangsu, China; ^3^Department of Cardiology, Northern Jiangsu People's Hospital, Yangzhou 225001, Jiangsu, China; ^4^Department of Ultrasound Medicine, Northern Jiangsu People's Hospital, Yangzhou 225001, Jiangsu, China

**Keywords:** contrast-induced acute kidney injury, coronary heart disease, percutaneous coronary intervention, predictive modeling

## Abstract

**Objective:** To identify risk factors for contrast-induced acute kidney injury (CI-AKI) post-PCI in coronary heart disease (CHD) patients, analyze novel inflammatory markers, and develop a predictive model.

**Methods:** CHD patients admitted to Northern Jiangsu People's Hospital in Yangzhou, Jiangsu Province, China, from January 1, 2019, to December 31, 2022, were selected, and a total of 628 patients were included in this study by collecting the general information, past history, and relevant laboratory test results of all patients and excluding those with imperfect relevant medical records, including 142 cases in the CI-AKI group and 486 cases in the non-CI-AKI group. According to the ratio of 7:3, they were randomly divided into a training group (*n* = 439) and a validation group (*n* = 189). Independent risk factors for the occurrence of postoperative CI-AKI were screened by unifactorial and multifactorial logistic regression analyses in the training group, a clinical prediction model was established, and the prediction efficiency and applicability of the prediction model were analyzed by ROC curves, DCA curves, and H–L curves in the two groups.

**Results:** Regression analysis suggested that neutrophil count, low-density lipoprotein, and PLR were independent risk factors for CI-AKI (*p* < 0.05); a model for predicting CI-AKI was established based on the above indexes, and the areas under the ROC curves of the model in the training and validation groups were 0.73 (0.67–0.78) and 0.71 (0.62–0.79), respectively; the H–L curve suggests that the predicted situation of the model is consistent with the actual occurrence, and the DCA curve suggests that patients in the training group and the validation group will have the greatest clinical benefit when the thresholds for the occurrence of postoperatively induced acute kidney injury are 0.26–0.82 and 0.30–0.97, respectively.

**Conclusion:** This CI-AKI prediction model demonstrates good accuracy and clinical applicability, aiding early high-risk patient identification and intervention.

**Trial Registration:** Chinese Registry of Clinical Trials: ChiCTR2500099751

## 1. Introduction

Coronary heart disease (CHD), a leading cause of global mortality and morbidity, imposes a significant burden on both the global economy and productivity, with its prevalence continuing to rise [1]. The pathogenesis of CHD is closely linked to atherosclerosis, a chronic inflammatory condition of the arterial system. Atherosclerosis is characterized by lipid deposition, inflammatory cell infiltration, and proliferation and fibrotic transformation of smooth muscle cells, leading to the development and progression of atherosclerotic plaques. As the disease progresses, these plaques enlarge, causing narrowing of the arterial lumen. This stenosis can trigger thrombus formation at the lesion site or lead to plaque rupture, resulting in downstream arterial thrombosis [2]. CHD occurs when the coronary arteries experience reduced blood flow or complete occlusion, primarily due to the detrimental effects of atherosclerosis.

Percutaneous coronary intervention (PCI) has become a preferred therapeutic strategy for CHD, providing rapid revascularization of occluded coronary arteries and significantly improving myocardial perfusion. However, the use of iodinated contrast media during PCI procedures can result in a clinically significant complication, known as contrast-induced acute kidney injury (CI-AKI). CI-AKI is characterized by a progressive decline in renal function, typically occurring within 48–72 h after contrast exposure. The underlying pathophysiological mechanisms primarily involve the direct cytotoxic effects of contrast agents on renal tubular epithelial cells, hemodynamic changes mediated by vasoactive substances, increased oxidative stress, and subsequent ischemic injury to the renal parenchyma [3]. According to the Chinese Expert Consensus on CI-AKI Diagnosis and Prevention, this condition is defined as either a relative increase in serum creatinine (SCr) levels by ≥ 25% or an absolute SCr elevation of ≥ 0.5 mg/dL (44.2 μmol/L) from baseline within 48–72 h postcontrast exposure, after excluding other causes of renal impairment [4]. Although CI-AKI is typically transient, with spontaneous recovery of creatinine levels within 1–3 weeks, it is independently associated with significantly higher risks of both short-term and long-term mortality [5].

In recent years, novel inflammatory markers, such as the neutrophil-to-lymphocyte ratio (NLR), platelet-to-lymphocyte ratio (PLR), and systemic immune–inflammatory index (SII), have gained significant attention as reliable biomarkers for monitoring systemic inflammatory responses in various pathological conditions, including malignancies, cardiovascular disorders, and SARS-CoV-2 infections [6]. These indices offer distinct advantages over traditional inflammatory markers, such as high-sensitivity C-reactive protein and erythrocyte sedimentation rate, due to their methodological simplicity, analytical stability, and cost-effectiveness. However, the potential association between these hematological indices and the risk of CI-AKI following PCI in patients with CHD remains poorly understood. This study aims to comprehensively evaluate eight underinvestigated inflammatory indices that have been either overlooked or excluded from previous predictive models. Using rigorous statistical analysis, we seek to (1) assess the predictive capacity of these novel biomarkers for CI-AKI development post-PCI in CHD patients and (2) construct an optimized predictive model. This investigation may provide valuable insights into early risk stratification and preventive strategies for CI-AKI, potentially improving clinical outcomes in this patient population.

## 2. Information and Methods

### 2.1. Research Subjects

This study retrospectively analyzed patients diagnosed with CHD who underwent PCI at the Department of Cardiology, Northern Jiangsu People's Hospital, Jiangsu, China, between January 1, 2019, and December 31, 2022. This study complied with the tenets of the Declaration of Helsinki. This retrospective study was approved by the Medical Ethics Committee of Northern Jiangsu People's Hospital (Approval number: 2025ky071), and retrospective studies exempt patients from informed consent.

Following rigorous screening based on predefined inclusion and exclusion criteria and ensuring data completeness, a total of 628 eligible patients were enrolled in the final analysis. Postoperative renal function was systematically evaluated through SCr measurements. Based on the diagnostic criteria for CI-AKI, the study population was stratified into two groups: (1) the CI-AKI group, comprising 142 patients who developed CI-AKI postprocedure, and (2) the non-CI-AKI group, consisting of 486 patients who maintained normal renal function following contrast exposure.

The inclusion criteria were as follows: (1) patients aged between 18 and 90 years; (2) absence of contraindications for PCI with successful completion of the procedure; and (3) availability of comprehensive clinical data, including complete pre- and postoperative laboratory results and procedural documentation.

**Figure 1:**
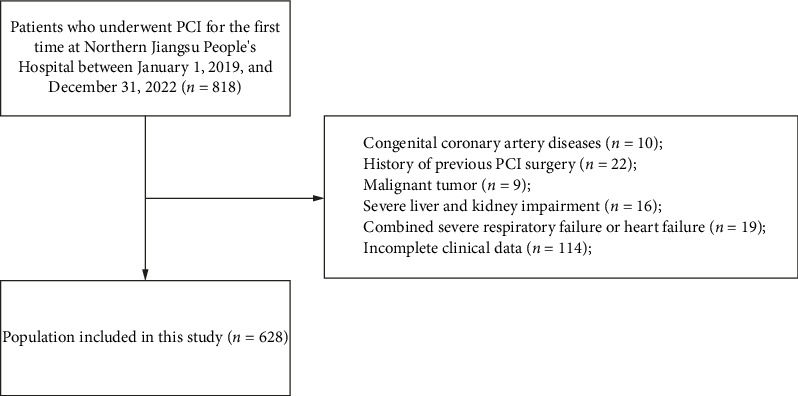
Flowchart of participant selection.

The exclusion criteria were as follows: (1) absence of SCr monitoring within 72 h post-PCI; (2) presence of severe hepatic dysfunction or advanced chronic kidney disease (CKD Stages 4–5), defined as glomerular filtration rate (GFR) < 30 mL/min/1.73 m^2^ [7]; (3) accompanied by New York Heart Association (NYHA) heart failure classification III–IV, long-term hypotension (systolic blood pressure [SBP] < 90 mmHg), cardiogenic shock, or long-term use of vasoactive drugs; (4) coexisting hyperthyroidism, active systemic infections, malignancies, autoimmune disorders, or hematological diseases; (5) received nephrotoxic drugs within 72 h before and after PCI; (6) documented history of contrast media hypersensitivity; and (7) recent contrast media exposure within 7 days preceding the PCI procedure (Figure 1).

### 2.2. Data Collection and Definitions

Clinical data were systematically extracted from the electronic medical record system, encompassing comprehensive demographic information, detailed medical history, and relevant laboratory parameters. The collected laboratory indices included complete blood count, lipid profile, fasting blood glucose, and hepatic and renal function tests. All laboratory measurements were obtained from the first fasting blood samples collected within 24 h of hospital admission.

Operational definitions and diagnostic criteria were defined as follows:

1. Smoking history: defined as regular consumption of > 1 cigarette per day, maintained for a minimum duration of 6 months or longer.
2. Alcohol consumption: categorized based on daily ethanol intake, with thresholds of ≥ 20 g/day for males and ≥ 10 g/day for females, consistent with World Health Organization guidelines.
3. Hypertension diagnosis: established when SBP ≥ 140 mmHg and/or diastolic blood pressure (DBP) ≥ 90 mmHg (1 mmHg = 0.133 kPa) was recorded on three separate occasions at nonconsecutive clinical visits [8].
4. Type 2 diabetes mellitus: diagnosed according to the most recent International Diabetes Federation (IDF) diagnostic criteria and classification guidelines [9].
5. Renal function stratification:
  Normal renal function: SCr < 133 μmol/L
  Compensated renal insufficiency: 133–176 μmol/L
  Decompensated renal insufficiency: 177–441 μmol/L
  Renal failure: 442–706 μmol/L
  Uremia: SCr ≥ 707 μmol/L
6. Inflammatory biomarker calculations:
  SII: (platelet count × neutrophil count)/lymphocyte count
  Systemic inflammation response index (SIRI): (neutrophil count × monocyte count)/lymphocyte count
  Aggregate index of systemic inflammation (AISI): (neutrophil count × platelet count × monocyte count)/lymphocyte count
  NLR: ratio of neutrophil count to lymphocyte count
  Lymphocyte-to-monocyte ratio (LMR): ratio of lymphocyte count to monocyte count
  PLR: ratio of platelet count to lymphocyte count
  Monocyte-to-high-density lipoprotein ratio (MHR): ratio of monocyte count to high-density lipoprotein cholesterol
  Neutrophil-to-high-density lipoprotein ratio (NHR): ratio of neutrophil count to high-density lipoprotein cholesterol

### 2.3. Study Methods

All study participants received standardized antiplatelet therapy according to current clinical guidelines prior to PCI, consisting of a loading dose of 300 mg aspirin combined with either 300 mg clopidogrel or 180 mg ticagrelor. Coronary angiography was performed following standardized procedural protocols by experienced interventional cardiologists. Quantitative coronary analysis was independently conducted by two board-certified interventional cardiologists, who assessed coronary artery stenosis severity through meticulous angiographic interpretation. The decision for PCI was made based on a comprehensive evaluation of multiple clinical parameters, including (1) characteristic angina symptoms, (2) electrocardiographic changes, (3) myocardial necrosis biomarkers, and (4) other relevant diagnostic findings. The intervention was specifically targeted at the identified culprit lesion(s). The nonionic, low-osmolar contrast agent iopromide (100 mL: 35 g iodine concentration; National Drug Code H20143027) was uniformly administered in all procedures, with dosage adjusted according to individual patient characteristics and procedural requirements.

### 2.4. Statistical Methods

Statistical analyses were conducted using SPSS (Version 27.0) and R software (Version 4.2.2). Continuous variables were tested for normality using the Shapiro–Wilk test and expressed as mean ± standard deviation ($\bar{x}$ ± *s*) if normally distributed, analyzed with the independent samples *t*-test; nonnormally distributed data were expressed as median with interquartile range [M (*Q*_1_, *Q*_3_)] and analyzed using the Mann–Whitney *U* test. Categorical variables were presented as frequencies (percentages) and compared using Pearson's *X*^2^ test or Fisher's exact test, as appropriate. Univariate logistic regression analysis was first performed to identify potential risk factors, followed by multivariate logistic regression analysis with backward stepwise selection to determine independent predictors of CI-AKI. The predictive performance of novel inflammatory indices for CI-AKI was evaluated using receiver operating characteristic (ROC) curve analysis, with the area under the curve (AUC) calculated to measure diagnostic accuracy. The “RMS” package in R software was used to develop a nomogram based on independent predictors identified through multivariate logistic regression analysis. Model performance was assessed using multiple metrics: (1) discrimination ability evaluated by AUC; (2) clinical utility assessed through decision curve analysis (DCA); and (3) calibration evaluated using the Hosmer–Lemeshow goodness-of-fit test and calibration plots. A two-tailed *p* value < 0.05 was considered statistically significant for all analyses.

## 3. Results

### 3.1. Comparison of Baseline Information Between the Training and Validation Groups

Based on the SCr results of preoperative and postoperative tests, the enrolled patients were divided into the CI-AKI group (*n* = 486) and the non-CI-AKI group (*n* = 142), and a ratio of 7:3 was chosen to divide all the study subjects into the training group (*n* = 439) and the validation group (*n* = 189). There was no significant abnormality in the comparison of general treatment and relevant laboratory test results between the patients in the two groups (*p* > 0.05), and the clinical characteristics of the patients in the two groups were consistent, as shown in Table 1.

### 3.2. Comparison of Information of the Population in the Training Group

In the training group (*n* = 439), the included patients were divided into the study group (*n* = 345) and the control group (*n* = 94) according to the presence or absence of CI-AKI. Comparison of the general data of the two groups of patients revealed that the number of patients with a history of diuretic use was significantly higher in the study group than in the control group, while the LVEF was lower than that in the control group (*p* < 0.05). Among the relevant laboratory tests, white blood cell count, neutrophil count, monocyte count, platelet count, fasting blood glucose, total cholesterol, low-density lipoprotein (LDL), SII, SIRI, AISI, M, NLR, PLR, and NHR were higher than those in the control group, while lymphocyte count and LMR were lower than those in the control group (*p* < 0.05), as detailed in Table 2.

### 3.3. Univariate and Multivariate Logistic Regression Analysis of the Occurrence of CI-AKI in the Training Group

Diuretic use, LVEF, white blood cell count, neutrophil count, monocyte count, platelet count, fasting blood glucose, total cholesterol, LDL, SII, SIRI, AISI, NLR, PLR, NHR, lymphocyte count, and LMR were used as independent variables for univariate logistic regression analysis. The results showed that diuretic use, white blood cells count, neutrophil count, monocyte count, platelet count, fasting blood glucose, total cholesterol, LDL, SII, SIRI, AISI, NLR, PLR, and NHR were independent risk factors for the occurrence of CI-AKI, and LVEF, lymphocyte count, and LMR were protective factors (*p* < 0.05), as shown in Table 3.

Further multifactorial logistic stepwise regression analysis was performed with the statistically significant indicators in the univariate logistic regression analysis as the independent variables, and the results showed that neutrophil count, LDL, and PLR were the independent risk factors for the occurrence of CI-AKI (*p* < 0.05), as shown in Table 4.

### 3.4. Nomogram Construction of CI-AKI Occurrence

**Figure 2:**
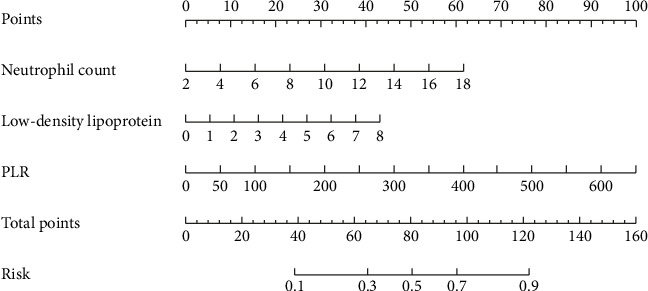
Nomogram construction of CI-AKI occurrence.

The nomogram predicting the occurrence of CI-AKI was constructed in the training group by incorporating the influencing factors screened in Table 4, as shown in Figure 2. The nomogram was used by first plotting each predictor variable's value vertically to the “Points” axis to obtain its corresponding score [10]. After summing all individual scores to derive the total points, a vertical projection from the “Total points” axis to the “Risk” axis indicated the patient's predicted likelihood of developing CI-AKI post-PCI.

### 3.5. Nomogram's Predicted Energy Efficiency Analysis in Training and Validation Groups

**Figure 3:**
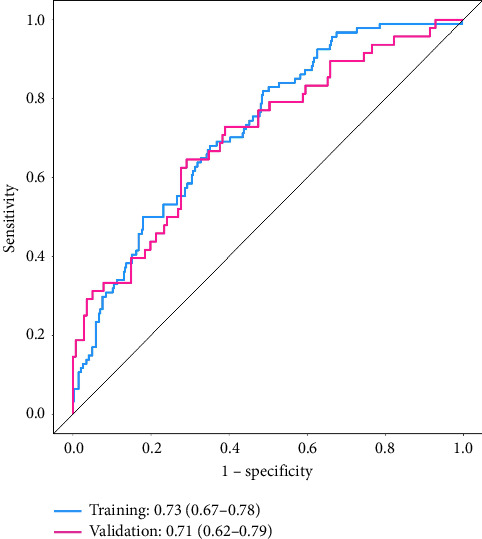
ROC curve analysis of the nomogram.

Analysis of the predicted energy efficiency of the nomogram in the training and validation groups by ROC curves revealed that the AUC value of the nomogram for predicting the occurrence of CI-AKI in patients in the training group was 0.73 (0.67–0.78) and that the AUC value of the nomogram in the validation group was 0.71 (0.62–0.79), as shown in Figure 3.

### 3.6. Nomogram Suitability Analysis

**Figure 4:**
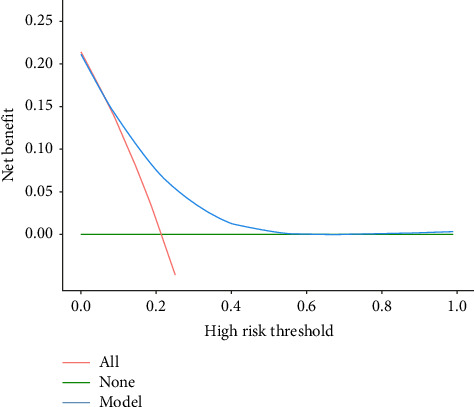
DCA curves for training group prediction models.

**Figure 5:**
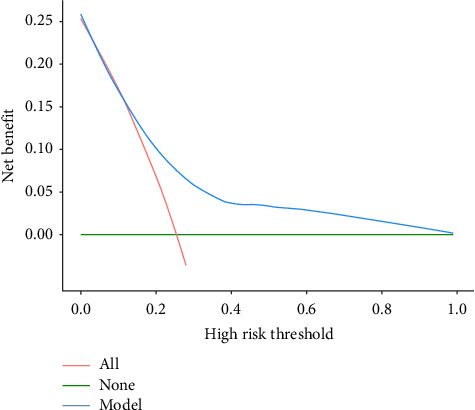
DCA curves for the validation group prediction model.

The DCA curve was used to analyze the applicability of the nomogram. The green “None” line represents no intervention for any patient, while the red “All” line indicates intervention for all patients. The blue “Model” line demonstrates the net benefit of our predictive model across the entire probability threshold spectrum. The DCA revealed that in both derivation (0.10–0.82) and validation (0.15–0.97) cohorts, the model's net benefit exceeded both the “All” and “None” strategies, confirming its practical clinical value, as shown in Figures 4 and 5.

### 3.7. Nomogram Predictive Efficiency Analysis

**Figure 6:**
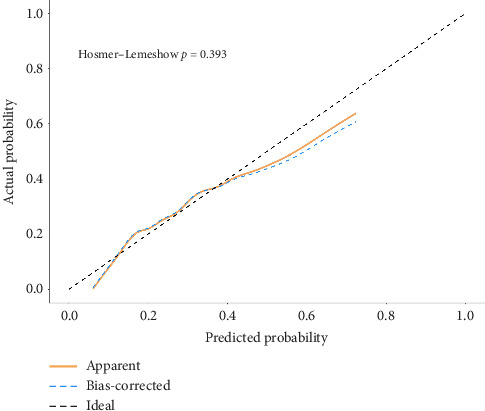
Calibration curve of the nomogram in the training group.

**Figure 7:**
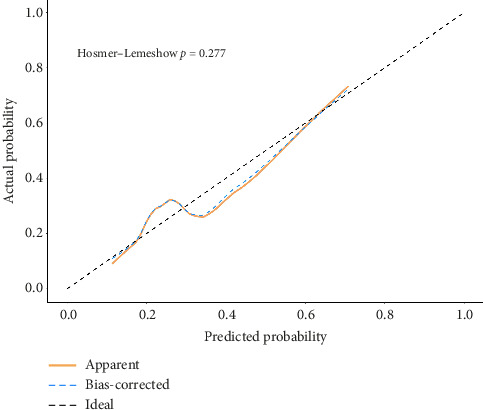
Calibration curve of the nomogram in the validation group.

The H–L deviance test found that in the training and validation groups, the differences between the predicted and actual occurrences of the two groups were not statistically significant (*p*=0.393, 0.277). As shown by the calibration curves of the training group and the validation group populations, there was good agreement between the predicted probability curves and the ideal curves of both the column-line diagram models, suggesting that the column-line diagrams had good calibration; see Figures 6 and 7.

## 4. Discussion

PCI has become the primary therapeutic approach for CHD, significantly improving patient prognosis. However, the occurrence of CI-AKI adversely affects postoperative recovery and leads to varying degrees of renal impairment in CHD patients. Despite substantial advancements in contrast agent development, their administration may trigger proinflammatory responses that directly contribute to renal tubular epithelial cell injury, increasing the risk of CI-AKI. To date, the precise pathogenesis of CI-AKI remains incompletely understood. Current evidence suggests that potential mechanisms may include contrast-induced disruption of vasodilatory regulation in the renal medulla, direct nephrotoxic effects of contrast media, secondary inflammatory cascades, and the acceleration of apoptotic pathways [11].

The multifactorial logistic regression analysis conducted in this study identified neutrophil count, LDL, and PLR as independent risk factors for CI-AKI in patients with CHD. Neutrophils, the most abundant circulating leukocytes, constitute approximately 50–70% of all leukocytes and exhibit extensive heterogeneity. These cells are among the first responders to sites of inflammation [12]. However, prolonged neutrophil infiltration can lead to tissue damage. Activated neutrophils play a crucial role in trapping and eliminating pathogenic bacteria through the release of neutrophil extracellular traps (NETs). When NETs are overproduced or not promptly cleared, this gelatinous network, rich in hydrolytic enzymes and DNA, adheres to the vascular endothelium, triggering endothelial cell apoptosis and subsequent tissue damage [13]. Furthermore, the release of NETs can exacerbate the progression of various renal diseases by promoting sterile necrotizing inflammation, thrombosis, and the production of anti-NET autoantibodies [14]. A study by Wang [15] provided further evidence that NETs are present in the kidneys of CI-AKI mice and contribute to disease progression by mediating endothelial cell involvement in the renal microcirculation. The study demonstrated that the removal of NETs significantly attenuated renal pathological injury and reduced SCr levels, highlighting the critical role of NETs in the pathogenesis of CI-AKI and suggesting potential therapeutic targets for mitigating this condition.

Dyslipidemia induces proatherosclerotic oxidative stress through the generation of oxidized lipids, which play a pivotal role in the pathogenesis of vascular inflammation and thrombotic events, ultimately contributing to the development of myocardial infarction, ischemic stroke, and deep vein thrombosis. The elevation of LDL, particularly its oxidized low-density lipoprotein (oxLDL), is recognized as a danger-associated molecular pattern (DAMP) that interacts with scavenger receptors, including LOX-1 and CD36. This interaction not only exacerbates vascular inflammation and accelerates atherosclerotic plaque formation but also enhances platelet activation while simultaneously suppressing platelet inhibitory pathways, thereby creating a prothrombotic state that significantly increases the risk of cardiovascular events [16]. Clinical investigations in patients with acute ST-segment elevation myocardial infarction (STEMI) undergoing primary PCI have demonstrated that elevated levels of oxLDL serve as an independent risk factor for CI-AKI [17]. These findings are in concordance with the results of the present study, further substantiating the detrimental impact of LDL, particularly its oxidized form, on renal function in post-PCI patients.

Among the eight novel inflammatory indicators evaluated in this study, PLR emerged as the sole statistically significant independent risk factor. As a robust inflammatory biomarker, PLR has demonstrated significant clinical utility across a spectrum of pathological conditions, including renal disorders, cardiovascular diseases, and malignant neoplasms [18]. The elevation of PLR typically results from a combination of increased platelet counts and decreased lymphocyte counts in peripheral circulation. Following myocardial injury or ischemic events, activation of the hypothalamic–pituitary–adrenal axis leads to elevated cortisol levels, which subsequently modulate leukocyte dynamics by inducing neutrophilia and lymphocytopenia [19]. Furthermore, the inflammatory cascade triggered by myocardial injury is characterized by microcirculatory disturbances, renal dysfunction, enhanced vascular permeability, and platelet activation, all of which contribute to further elevation of PLR and exacerbate systemic inflammation [20]. Clinical evidence indicates that acute myocardial infarction (AMI) patients with elevated PLR exhibit worse in-hospital outcomes, suggesting that PLR serves as an independent predictor of hospital-acquired complications. The underlying mechanisms likely involve a synergistic interplay between heightened inflammatory responses and a prothrombotic state, which collectively contribute to adverse clinical outcomes in patients with elevated PLR [21].

## 5. Conclusion

In this study, three independent influencing factors were screened by univariate and multivariate logistic regression analysis, and this was used to construct a risk prediction model applicable to post-PCI CI-AKI in CHD patients of East China's Jiangsu Province. The model was presented in the form of a nomogram graph, providing an intuitive scoring system for assessing the risk of developing CI-AKI after PCI. The results of the model validation showed that the AUC of the working characteristics of the subjects in both the modeling and validation groups was greater than 0.70, and the results of the analysis of the decision curves in the two groups were in high concordance, suggesting that the model has wide applicability in clinical decision-making and can provide clinical benefits for most patients. The predictors involved in this model are all routine clinical data of patients during hospitalization, without additional testing or complex calculations, which significantly improves its clinical utility and operability.

The current study has several important limitations that should be acknowledged. First, as a single-center retrospective analysis with a relatively modest sample size and exclusion of patients with incomplete data, the findings may be subject to selection bias and limited generalizability to broader populations. Second, while internal validation demonstrated good model performance, the lack of external validation leaves the model's applicability to other clinical settings uncertain. To address these limitations, our future research will focus on (1) conducting multicenter prospective studies with larger, more representative samples to enhance the model's robustness; (2) performing external validation across diverse clinical settings; and (3) undertaking head-to-head comparisons against established risk scores to rigorously evaluate the model's incremental clinical value and potential for widespread implementation.

## Figures and Tables

**Table 1:** Comparison of information between the training and validation groups.

Variables	Total (*n* = 628)	Validation (*n* = 189)	Training (*n* = 439)	Statistic	*p*
Age [M (*Q*_1_, *Q*_3_), years]	67.00 (56.00, 78.00)	67.00 (55.00, 79.00)	67.00 (56.00, 78.00)	*Z* = −0.52	0.604
LVEF [M (*Q*_1_, *Q*_3_), (%)]	52.44 (52.44, 55.00)	52.44 (52.44, 54.00)	52.44 (52.44, 55.00)	*Z* = −0.66	0.509
Gender [*n*, (%)]				*χ* ^2^ = 0.33	0.568
Female	125 (19.90)	35 (18.52)	90 (20.50)		
Male	503 (80.10)	154 (81.48)	349 (79.50)		
Hypertension [*n*, (%)]				*χ* ^2^ = 0.30	0.582
No	239 (38.06)	75 (39.68)	164 (37.36)		
Yes	389 (61.94)	114 (60.32)	275 (62.64)		
Atrial fibrillation [*n*, (%)]				*χ* ^2^ = 2.36	0.124
No	589 (93.79)	173 (91.53)	416 (94.76)		
Yes	39 (6.21)	16 (8.47)	23 (5.24)		
Diabetes [*n*, (%)]				*χ* ^2^ = 0.01	0.908
No	434 (69.11)	130 (68.78)	304 (69.25)		
Yes	194 (30.89)	59 (31.22)	135 (30.75)		
Smoking [*n*, (%)]				*χ* ^2^ = 0.70	0.401
No	260 (41.40)	83 (43.92)	177 (40.32)		
Yes	368 (58.60)	106 (56.08)	262 (59.68)		
Drinking [*n*, (%)]				*χ* ^2^ = 0.01	0.943
No	526 (83.76)	158 (83.60)	368 (83.83)		
Yes	102 (16.24)	31 (16.40)	71 (16.17)		
Perioperative drugs [*n*, (%)]					
Diuretics [*n*, (%)]				*χ* ^2^ = 1.93	0.164
No	503 (80.10)	145 (76.72)	358 (81.55)		
Yes	125 (19.90)	44 (23.28)	81 (18.45)		
ACEI/ARB [*n*, (%)]				*χ* ^2^ = 0.44	0.509
No	476 (75.80)	140 (74.07)	336 (76.54)		
Yes	152 (24.20)	49 (25.93)	103 (23.46)		
Beta-blockers [*n*, (%)]				*χ* ^2^ = 0.13	0.718
No	276 (43.95)	81 (42.86)	195 (44.42)		
Yes	352 (56.05)	108 (57.14)	244 (55.58)		
Diabetes drug [*n*, (%)]				*χ* ^2^ = 0.04	0.836
No	455 (72.45)	138 (73.02)	317 (72.21)		
Yes	173 (27.55)	51 (26.98)	122 (27.79)		
Contrast agent dosage [M (*Q*_1_, *Q*_3_), mL]	245.50 (200.00, 296.00)	242.00 (204.00, 287.00)	247.00 (199.00, 299.50)	*Z* = −0.57	0.570
Coronary lesion site [*n*, (%)]				*χ* ^2^ = 0.12	0.989
LAD	167 (26.59)	51 (26.98)	116 (26.42)		
LCX	160 (25.48)	48 (25.40)	112 (25.51)		
LM	148 (23.57)	43 (22.75)	105 (23.92)		
RCA	153 (24.36)	47 (24.87)	106 (24.15)		
Hemoglobin [M (*Q*_1_, *Q*_3_), g/L]	136.00 (124.00, 148.00)	134.40 (124.00, 148.00)	136.00 (124.00, 149.00)	*Z* = −0.90	0.369
White blood cell count [M (*Q*_1_, *Q*_3_), × 10^9^/L]	8.81 (7.13, 10.97)	8.67 (7.18, 10.55)	8.85 (7.08, 11.14)	*Z* = −1.11	0.269
Neutrophil count [M (*Q*_1_, *Q*_3_), × 10^9^/L]	6.46 (4.95, 8.21)	6.39 (5.12, 8.08)	6.50 (4.92, 8.43)	*Z* = −0.63	0.526
Lymphocyte count [M (*Q*_1_, *Q*_3_), × 10^9^/L]	1.57 (1.23, 1.99)	1.59 (1.16, 1.96)	1.57 (1.27, 2.00)	*Z* = −1.09	0.276
Monocyte count [M (*Q*_1_, *Q*_3_), × 10^9^/L]	0.59 (0.44, 0.76)	0.57 (0.45, 0.70)	0.60 (0.44, 0.79)	*Z* = −1.81	0.070
Platelet count [M (*Q*_1_, *Q*_3_), × 10^9^/L]	186.50 (152.00, 223.00)	186.00 (152.00, 218.00)	187.00 (152.00, 224.00)	*Z* = −0.37	0.711
Fasting blood glucose [M (*Q*_1_, *Q*_3_), mmol/L]	6.21 (5.09, 7.57)	6.42 (5.18, 7.90)	6.15 (5.06, 7.50)	*Z* = −0.92	0.359
Glycosylated hemoglobin, type A1C [M (*Q*_1_, *Q*_3_), %]	6.80 (5.90, 6.80)	6.80 (5.90, 6.80)	6.80 (5.90, 6.80)	*Z* = −0.10	0.923
Triglycerides [M (*Q*_1_, *Q*_3_), mmol/L]	1.59 (1.13, 2.29)	1.61 (1.10, 2.36)	1.57 (1.14, 2.24)	*Z* = −0.24	0.809
Total cholesterol [M (*Q*_1_, *Q*_3_), mmol/L]	4.34 (3.72, 4.91)	4.37 (3.74, 4.90)	4.28 (3.67, 4.92)	*Z* = −0.57	0.565
High-density lipoprotein [M (*Q*_1_, *Q*_3_), mmol/L]	1.03 (0.86, 1.22)	1.03 (0.83, 1.21)	1.03 (0.86, 1.22)	*Z* = −0.41	0.684
Low-density lipoprotein [M (*Q*_1_, *Q*_3_), mmol/L]	2.71 (2.21, 3.22)	2.74 (2.26, 3.23)	2.69 (2.17, 3.22)	*Z* = −0.76	0.446
Lipoprotein a [M (*Q*_1_, *Q*_3_), g/L]	196.10 (120.45, 288.33)	184.90 (124.90, 262.90)	202.20 (118.80, 292.65)	*Z* = −0.45	0.652
Apolipoprotein A1 [M (*Q*_1_, *Q*_3_), g/L]	1.20 (1.06, 1.34)	1.17 (1.06, 1.33)	1.20 (1.07, 1.35)	*Z* = −1.04	0.297
Apolipoprotein B [M (*Q*_1_, *Q*_3_), g/L]	0.97 (0.82, 1.14)	0.98 (0.86, 1.13)	0.97 (0.80, 1.15)	*Z* = −1.11	0.266
Albumin [M (*Q*_1_, *Q*_3_), g/L]	39.60 (37.00, 42.23)	39.60 (37.00, 42.20)	39.60 (37.05, 42.25)	*Z* = −0.02	0.987
Alanine aminotransferase [M (*Q*_1_, *Q*_3_), U/L]	39.00 (26.75, 55.00)	38.00 (28.00, 52.00)	39.00 (26.00, 56.00)	*Z* = −0.40	0.691
Aspartate aminotransferase [M (*Q*_1_, *Q*_3_), U/L]	57.00 (37.00, 80.00)	56.00 (36.00, 81.00)	58.00 (37.00, 79.50)	*Z* = −0.45	0.656
Potassium [M (*Q*_1_, *Q*_3_), mmol/L]	3.85 (3.62, 4.11)	3.86 (3.63, 4.17)	3.85 (3.62, 4.08)	*Z* = −0.81	0.420
Uric acid [M (*Q*_1_, *Q*_3_), μmol/L]	319.00 (260.20, 374.00)	316.80 (260.60, 362.00)	320.30 (260.00, 376.45)	*Z* = −1.36	0.174
Preoperative creatinine [M (*Q*_1_, *Q*_3_), μmol/L]	80.00 (69.15, 91.00)	77.00 (68.20, 89.45)	81.00 (70.00, 92.00)	*Z* = −1.42	0.154
C-reactive protein [M (*Q*_1_, *Q*_3_), mg/L]	16.00 (5.78, 29.00)	15.00 (4.97, 28.68)	17.00 (6.00, 29.00)	*Z* = −0.97	0.330
Post-operative creatinine [M (*Q*_1_, *Q*_3_), μmol/L]	96.00 (79.00, 96.24)	95.00 (80.00, 96.24)	96.00 (79.00, 96.24)	*Z* = −0.08	0.936
SII [M (*Q*_1_, *Q*_3_)]	741.65 (494.76, 1061.79)	736.39 (499.91, 1050.14)	744.55 (494.57, 1066.37)	*Z* = −0.00	1.000
SIRI [M (*Q*_1_, *Q*_3_)]	2.41 (1.51, 3.64)	2.42 (1.54, 3.42)	2.40 (1.45, 3.81)	*Z* = −0.45	0.653
AISI [M (*Q*_1_, *Q*_3_)]	430.33 (265.39, 706.52)	427.23 (282.21, 639.23)	435.55 (264.44, 736.99)	*Z* = −0.87	0.382
NLR [M (*Q*_1_, *Q*_3_)]	3.94 (2.97, 5.67)	3.98 (2.99, 5.72)	3.91 (2.94, 5.62)	*Z* = −0.72	0.471
LMR [M (*Q*_1_, *Q*_3_)]	2.71 (2.04, 3.57)	2.71 (2.17, 3.52)	2.70 (1.99, 3.65)	*Z* = −0.22	0.829
PLR [M (*Q*_1_, *Q*_3_)]	116.56 (91.61, 155.25)	116.33 (92.11, 159.13)	116.67 (90.55, 152.62)	*Z* = −0.60	0.547
MHR [M (*Q*_1_, *Q*_3_)]	0.56 (0.39, 0.79)	0.55 (0.39, 0.74)	0.57 (0.39, 0.81)	*Z* = −1.20	0.230
NHR [M (*Q*_1_, *Q*_3_)]	6.23 (4.55, 8.60)	6.19 (4.66, 8.29)	6.23 (4.48, 8.66)	*Z* = −0.36	0.721

*Note:* Z: Mann–Whitney test; *χ*^2^: chi-square test; M: median; *Q*_1_: 1st quartile, *Q*_3_: 3rd quartile, LCX: left circumflex.

Abbreviations: ACEI/ARB, angiotensin-converting enzyme inhibitor/angiotensin II receptor blocker; LAD, left anterior descending; LM, left main; LVEFs, left ventricular ejection fractions; RCA, right coronary artery.

**Table 2:** Comparison of information between the study and control groups.

Variables	Total (*n* = 439)	Control group (*n* = 345)	Study group (*n* = 94)	Statistic	*p*
Age [M (*Q*_1_, *Q*_3_), years]	67.00 (56.00, 78.00)	68.00 (57.00, 78.00)	66.00 (56.00, 75.00)	*Z* = −1.22	0.224
LVEF [M (*Q*_1_, *Q*_3_), (%)]	52.44 (52.44, 55.00)	52.44 (52.44, 56.00)	52.44 (48.00, 54.00)	*Z* = −2.63	0.008
Gender [*n*, (%)]				*χ* ^2^ = 0.04	0.834
No	90 (20.50)	70 (20.29)	20 (21.28)		
Yes	349 (79.50)	275 (79.71)	74 (78.72)		
Hypertension [*n*, (%)]				*χ* ^2^ = 0.05	0.832
No	164 (37.36)	128 (37.10)	36 (38.30)		
Yes	275 (62.64)	217 (62.90)	58 (61.70)		
Atrial fibrillation [*n*, (%)]				*χ* ^2^ = 0.05	0.824
No	416 (94.76)	326 (94.49)	90 (95.74)		
Yes	23 (5.24)	19 (5.51)	4 (4.26)		
Diabetes [*n*, (%)]				*χ* ^2^ = 1.07	0.302
No	304 (69.25)	243 (70.43)	61 (64.89)		
Yes	135 (30.75)	102 (29.57)	33 (35.11)		
Smoking [*n*, (%)]				*χ* ^2^ = 0.25	0.618
No	177 (40.32)	137 (39.71)	40 (42.55)		
Yes	262 (59.68)	208 (60.29)	54 (57.45)		
Drinking [*n*, (%)]				*χ* ^2^ = 1.02	0.312
No	368 (83.83)	286 (82.90)	82 (87.23)		
Yes	71 (16.17)	59 (17.10)	12 (12.77)		
Perioperative drugs					
Diuretics [*n*, (%)]				*χ* ^2^ = 5.27	0.022
No	358 (81.55)	289 (83.77)	69 (73.40)		
Yes	81 (18.45)	56 (16.23)	25 (26.60)		
ACEI/ARB [*n*, (%)]				*χ* ^2^ = 0.70	0.402
No	336 (76.54)	261 (75.65)	75 (79.79)		
Yes	103 (23.46)	84 (24.35)	19 (20.21)		
Beta-blockers [*n*, (%)]				*χ* ^2^ = 0.03	0.860
No	195 (44.42)	154 (44.64)	41 (43.62)		
Yes	244 (55.58)	191 (55.36)	53 (56.38)		
Diabetes drug [*n*, (%)]				*χ* ^2^ = 0.56	0.455
No	317 (72.21)	252 (73.04)	65 (69.15)		
Yes	122 (27.79)	93 (26.96)	29 (30.85)		
Contrast agent dosage [M (*Q*_1_, *Q*_3_), mL]	247.00 (199.00, 299.50)	246.00 (197.00, 302.00)	251.50 (203.00, 289.50)	*Z* = −0.00	0.999
Criminal vessels [*n*, (%)]				*χ* ^2^ = 5.14	0.162
LAD	116 (26.42)	85 (24.64)	31 (32.98)		
LCX	112 (25.51)	94 (27.25)	18 (19.15)		
LM	105 (23.92)	86 (24.93)	19 (20.21)		
RCA	106 (24.15)	80 (23.19)	26 (27.66)		
Hemoglobin [M (*Q*_1_, *Q*_3_), g/L]	136.00 (124.00, 149.00)	135.00 (124.00, 147.00)	139.00 (127.00, 152.00)	*Z* = −1.55	0.122
White blood cell count [M (*Q*_1_, *Q*_3_), × 10^9^/L]	8.85 (7.08, 11.14)	8.58 (6.88, 10.72)	10.32 (8.07, 11.88)	*Z* = −3.90	< 0.001
Neutrophil count [M (*Q*_1_, *Q*_3_), × 10^9^/L]	6.50 (4.92, 8.43)	6.20 (4.79, 7.85)	7.50 (6.10, 9.80)	*Z* = −4.83	< 0.001
Lymphocyte count [M (*Q*_1_, *Q*_3_), × 10^9^/L]	1.57 (1.27, 2.00)	1.57 (1.28, 2.05)	1.48 (1.18, 1.85)	*Z* = −2.09	0.036
Monocyte count [M (*Q*_1_, *Q*_3_), × 10^9^/L]	0.60 (0.44, 0.79)	0.59 (0.43, 0.77)	0.65 (0.47, 0.84)	*Z* = −2.05	0.041
Platelet count [M (*Q*_1_, *Q*_3_), × 10^9^/L]	187.00 (152.00, 224.00)	182.00 (150.00, 215.00)	204.50 (172.25, 242.00)	*Z* = −3.60	< 0.001
Fasting blood glucose [M (*Q*_1_, *Q*_3_), mmol/L]	6.15 (5.06, 7.50)	5.95 (4.98, 7.17)	6.79 (5.57, 8.17)	*Z* = −2.92	0.004
Glycosylated hemoglobin, type A1C [M (*Q*_1_, *Q*_3_), %]	6.80 (5.90, 6.80)	6.80 (5.90, 6.80)	6.80 (5.82, 7.20)	*Z* = −0.38	0.705
Triglycerides [M (*Q*_1_, *Q*_3_), mmol/L]	1.57 (1.14, 2.24)	1.55 (1.13, 2.30)	1.63 (1.16, 2.14)	*Z* = −0.26	0.797
Total cholesterol [M (*Q*_1_, *Q*_3_), mmol/L]	4.28 (3.67, 4.92)	4.17 (3.58, 4.88)	4.44 (3.97, 5.12)	*Z* = −2.42	0.016
High-density lipoprotein [M (*Q*_1_, *Q*_3_), mmol/L]	1.03 (0.86, 1.22)	1.02 (0.86, 1.22)	1.03 (0.88, 1.23)	*Z* = −0.74	0.459
Low-density lipoprotein [M (*Q*_1_, *Q*_3_), mmol/L]	2.69 (2.17, 3.22)	2.63 (2.08, 3.17)	2.80 (2.40, 3.43)	*Z* = −2.77	0.006
Lipoprotein a [M (*Q*_1_, *Q*_3_), g/L]	202.20 (118.80, 292.65)	203.80 (120.30, 285.10)	194.95 (114.12, 342.82)	*Z* = −0.49	0.624
Apolipoprotein A1 [M (*Q*_1_, *Q*_3_), g/L]	1.20 (1.07, 1.35)	1.20 (1.06, 1.34)	1.24 (1.08, 1.42)	*Z* = −1.62	0.105
Apolipoprotein B [M (*Q*_1_, *Q*_3_), g/L]	0.97 (0.80, 1.15)	0.96 (0.78, 1.13)	0.98 (0.86, 1.17)	*Z* = −1.90	0.058
Albumin [M (*Q*_1_, *Q*_3_),g/L]	39.60 (37.05, 42.25)	39.40 (37.10, 42.20)	40.00 (36.65, 42.90)	*Z* = −0.66	0.508
Alanine aminotransferase [M (*Q*_1_, *Q*_3_), U/L]	39.00 (26.00, 56.00)	38.00 (25.00, 56.00)	41.00 (29.25, 58.75)	*Z* = −1.23	0.218
Aspartate aminotransferase [M (*Q*_1_, *Q*_3_), U/L]	58.00 (37.00, 79.50)	56.00 (37.00, 80.00)	62.50 (38.00, 76.75)	*Z* = −0.25	0.803
Potassium [M (*Q*_1_, *Q*_3_), mmol/L]	3.85 (3.62, 4.08)	3.85 (3.62, 4.07)	3.78 (3.61, 4.10)	*Z* = −0.35	0.728
Uric acid [M (*Q*_1_, *Q*_3_), μmol/L]	320.30 (260.00, 376.45)	321.00 (268.00, 376.00)	302.50 (241.80, 374.42)	*Z* = −0.99	0.324
C-reactive protein [M (*Q*_1_, *Q*_3_), mg/L]	17.00 (6.00, 29.00)	17.00 (5.76, 29.00)	17.50 (7.00, 29.75)	*Z* = −0.33	0.742
Renal function classification [*n*, (%)]				*χ* ^2^ = 1.075	0.584
Normal kidney function	424 (96.58)	333 (96.52)	91 (96.81)		
Compensated renal function	9 (2.05)	8 (2.32)	1 (1.06)		
Uncompensated kidney function	6 (1.37)	4 (1.16)	2 (2.13)		
SII [M (*Q*_1_, *Q*_3_)]	744.55 (494.57, 1066.37)	661.66 (457.23, 972.00)	1048.87 (758.26, 1521.75)	*Z* = −6.60	< 0.001
SIRI [M (*Q*_1_, *Q*_3_)]	2.40 (1.45, 3.81)	2.22 (1.34, 3.45)	2.95 (2.06, 5.28)	*Z* = −4.85	< 0.001
AISI [M (*Q*_1_, *Q*_3_)]	435.55 (264.44, 736.99)	394.19 (234.11, 633.22)	670.97 (423.04, 1063.15)	*Z* = −5.83	< 0.001
NLR [M (*Q*_1_, *Q*_3_)]	3.91 (2.94, 5.62)	3.77 (2.66, 5.12)	5.16 (3.50, 6.89)	*Z* = −5.33	< 0.001
LMR [M (*Q*_1_, *Q*_3_)]	2.70 (1.99, 3.65)	2.79 (2.13, 3.76)	2.39 (1.66, 3.30)	*Z* = −3.67	< 0.001
PLR [M (*Q*_1_, *Q*_3_)]	116.67 (90.55, 152.62)	111.18 (84.29, 143.81)	136.04 (106.67, 179.46)	*Z* = −4.61	< 0.001

**Table 3:** Univariate logistic regression analysis of CI-AKI occurrence.

Variables	*β*	SE	Z	*p*	OR (95% CI)
Diuretics	0.626	0.275	2.273	0.023	1.870 (1.090∼3.208)
LVEF	−0.044	0.017	−2.516	0.012	0.957 (0.925∼0.990)
White blood cell count	0.156	0.040	3.858	< 0.001	1.169 (1.080∼1.265)
Neutrophil count	0.217	0.045	4.802	< 0.001	1.242 (1.137∼1.357)
Lymphocyte count	−0.488	0.204	−2.399	0.016	0.614 (0.412∼0.915)
Monocyte count	0.940	0.430	2.187	0.029	2.560 (1.102∼5.945)
Platelet count	0.006	0.002	3.303	< 0.001	1.006 (1.003∼1.010)
Fasting blood glucose	0.094	0.038	2.501	0.012	1.099 (1.021∼1.183)
Total cholesterol	0.212	0.105	2.013	0.044	1.236 (1.006∼1.519)
Low-density lipoprotein	0.332	0.123	2.697	0.007	1.394 (1.095∼1.774)
SII	0.001	0.000	5.642	< 0.001	1.001 (1.001∼1.002)
SIRI	0.232	0.048	4.852	< 0.001	1.261 (1.148∼1.385)
AISI	0.001	0.000	5.333	< 0.001	1.001 (1.001∼1.002)
NLR	0.193	0.042	4.612	< 0.001	1.213 (1.117∼1.317)
LMR	−0.362	0.109	−3.320	< 0.001	0.696 (0.562∼0.862)
PLR	0.009	0.002	4.268	< 0.001	1.009 (1.005∼1.013)
NHR	0.112	0.034	3.306	< 0.001	1.119 (1.047∼1.195)

**Table 4:** Multifactorial logistic regression analysis of CI-AKI occurrence.

Variables	*β*	SE	Z	*p*	OR (95% CI)
Neutrophil count	0.204	0.046	4.398	< 0.001	1.227 (1.120∼1.344)
Low-density lipoprotein	0.285	0.128	2.228	0.026	1.330 (1.035∼1.709)
PLR	0.008	0.002	3.870	< 0.001	1.008 (1.004∼1.012)

## Data Availability

The data that support the findings of this study are available from the corresponding author upon reasonable request.
